# Mitotherapy and the Possibility of Energetic Rescue in Evolving Brain Death

**DOI:** 10.5812/ijpr-168337

**Published:** 2025-12-21

**Authors:** Ali Dabbagh, Jalal Pourahmad, Amir Farrokhian

**Affiliations:** 1 Department of Anesthesiology, Anesthesiology Research Center, School of Medicine, Shahid Beheshti University of Medical Sciences, Tehran, Iran; 2 Department of Toxicology and Pharmacology, School of Pharmacy, Shahid Beheshti University of Medical Sciences Tehran, Iran; 3 Department of Clinical Pharmacy, School of Pharmacy, Shahid Beheshti University of Medical Sciences, Tehran, Iran

**Keywords:** Mitochondrial Therapy, Brain Death, Mitotherapy, Mitochondria

*Dear Editor,*

Brain death is defined as the irreversible cessation of all brain functions; however, the biological sequence that leads to this brain death is not yet fully understood. Accumulating evidence suggests that what we currently label as “irreversibility” is largely the consequence of a profound bioenergetic collapse — an event in which mitochondrial failure, rather than structural injury alone, determines the terminal loss of neuronal viability. This perspective opens a provocative question: If the critical determinant of irreversibility is energetic failure, could mitochondrial-based interventions alter the trajectory before brain death becomes fixed (1-3)? 

Ischemia is the primary trigger causing catastrophic damage to the mitochondrial architecture in the central nervous system. During acute cerebral hypoxia-ischemia, mitochondrial oxygen consumption decreases within minutes, the membrane action potential collapses, and ATP synthesis is rapidly reduced (4). Impaired electron transport accelerates the production of reactive oxygen species, causing lipid peroxidation, protein oxidation, and mitochondrial permeability transition; as cytochrome c escapes into the cytosol, the intrinsic apoptotic cascade is activated (5). But from here on, what happens is not cell death, but a rapidly expanding energetic vacuum: If mitochondria become dysfunctional, cellular ion homeostasis breaks down, membrane pumps fail, and neurons lose the ability to maintain structural and electrical integrity. In severe global ischemia, this cascade spreads across brain regions and culminates in the metabolic silence characteristic of brain death (4, 6).

Current neuroprotective strategies are inadequate because they fail to directly address this energy collapse. Hemodynamic optimization, oxygenation, osmotherapy, and hypothermia may reduce secondary damage, but none restore mitochondrial function after the respiratory chain has failed. This therapeutic gap, which is in fact a paradox between the initial injury and the treatment methods and is the source of irreversible damage, is precisely where mitotherapy may play a role as a potentially life-saving concept (7, 8).

Mitochondrial targeted therapy (mitotherapy) encompasses several approaches aimed at restoring or enhancing mitochondrial function (9). The most important of these approaches include: Direct mitochondrial transplantation, stimulation of endogenous mitochondrial biogenesis, transfer of healthy mitochondria via extracellular vesicles, mitochondrial-targeted peptides, and modulation of intercellular mitochondrial exchange. Experimental data are still preliminary and require further study; however, these initial research findings are very promising. In animal models of cerebral ischemia, exogenous mitochondria delivered to damaged brain tissue have shown rapid internalization by neurons and glia, restoration of ATP levels, increased oxygen consumption, reduced neuronal apoptosis, and improved functional outcomes (10-12).

Mitochondrial transfer from astrocytes to neurons has been observed as a natural salvage mechanism, suggesting an intrinsic biological background for mitochondrial replacement (13, 14). These findings lend mechanistic validity to the idea that restoration of bioenergetic capacity, even after profound injury, may revive cellular processes once thought to be permanently shut down.
